# Efficacy of Injectable Platelet-Rich Fibrin for Interdental Papilla Reconstruction Compared With Connective Tissue Grafts: A Randomized Controlled Clinical Trial

**DOI:** 10.7759/cureus.99017

**Published:** 2025-12-11

**Authors:** Jeswin Johnson, Baiju RM, Tony Kurien J, Neethu P Reghu, Rekha P Radhakrishnan, Shijna Ashraf, Santhosh Kumar S

**Affiliations:** 1 Department of Periodontics, Government Dental College, Kottayam, Kottayam, IND

**Keywords:** autologous platelet concentrates, gingival black triangle, interdental papilla recession, interdental papillary reconstruction, i-prf regeneration, papillary augmentation, periodontal regeneration, perio-esthetics

## Abstract

Introduction

The loss of interdental papilla in the anterior region of the mouth results in aesthetic and functional impairment. To overcome the limitations of soft-tissue grafts, autogenous platelet concentrates have been developed and shown promising results for periodontal regeneration. Injectable platelet-rich fibrin (I-PRF) slowly releases growth factors and can enhance neovascularization. The present study compared the clinical efficacy of I-PRF with that of connective tissue grafts (CTGs) in the treatment of interdental papilla deficits.

Materials and methods

Patients with class I or II papilla defect sites (based on the Nordland and Tarnow classification) in the maxillary anterior region were randomly assigned (n = 15) to either the test group, the members of which were treated with I-PRF microneedling (the I-PRF group), or the control group, the members of which were treated with CTGs using the tunneling technique (the CTG group). The full-mouth plaque score, the full-mouth bleeding score, the probing pocket depth, the clinical attachment level, the interdental papilla height (IDPH), and the black triangle height (BTH) were recorded at baseline and at one month and three months after the intervention. The patients provided self-recorded visual analog scale (VAS) scores on the third and seventh postoperative days. Intra- and inter-group comparisons were made using nonparametric tests.

Results

Both groups showed significant increases in IDPH and reductions in BTH from the baseline to three months. The intra-group analysis showed a significant difference in the IDPH and BTH values between the evaluation periods, whereas the inter-group comparison was not significant. The VAS scores for the I-PRF group were less than those for the CTG group.

Conclusions

I-PRF microneedling was comparable to CTGs using the tunneling technique in improving papillary dimensions in patients with interdental papilla deficiency. Microneedle administration of noninvasive I-PRF offers the advantages of patient comfort and less morbidity.

## Introduction

Growing concern about aesthetics among the general population is driving the focus on this issue in current dental practice. Interdental papilla deficiency, known as “black triangles,” is the third most common aesthetic concern in this regard [1]. Apart from its aesthetic implications, this deficiency is associated with food impaction and subsequent impairment of periodontal health [2].

A common cause of interdental papilla loss is destruction of the interproximal bone as a consequence of periodontitis. Black triangles can also arise as a complication following treatment for periodontitis [3]. Other factors, such as the gingival biotype, the gingival angle, the bone crest to interproximal contact point, and the interproximal distance between adjacent teeth, determine the extent of embrasure fill [3,4]. Black triangles become more prevalent with age, with a 67% increase among individuals over 20 years old [2]. Various classification systems have been proposed to evaluate interdental papilla loss, which have proved helpful in determining prognosis and subsequent treatment [5,6].

Many treatment strategies, both nonsurgical and surgical, have been developed to mitigate interdental papilla loss [7]. Various flap-based regenerative surgical procedures are the treatment of choice when the loss is associated with periodontal diseases [4]. In situations in which papilla deficiency is present in a healthy periodontium, therapeutic goals can be achieved by targeting the dentition through restorative or orthodontic treatment or soft tissue augmentation methods [8]. Traditionally, the surgical procedure for papilla regeneration has involved connective tissue grafts (CTGs), which can increase tissue thickness and stability. However, such flap-based procedures compromise tissue vascularity.

Currently, the primary medical and surgical interventions are minimally invasive. Hence, tunnel-modified techniques have been introduced to maintain the blood supply, which is a crucial factor in graft survival and clinical predictability. Feuillet et al. proposed using the interproximal tunneling technique in conjunction with customized CTGs. They reported favorable results in papilla gain, while a second surgical site associated with these grafts and increased patient morbidity led the clinicians to identify soft tissue substitutes [9].

Hyaluronic acid is a tissue filler extensively used to manage a receded papilla [10]. McGuire and Scheyer conducted a randomized controlled trial comparing the injection of autologous fibroblasts with a placebo. They found that the treatment group showed significant increases in papillary height at the two-month follow-up examination [11]. Recently, platelet concentrates, an autologous derivative, have shown promising results in papilla regeneration, mainly due to enhanced neovascularization. The highly polymerized three-dimensional fibrin network in platelet-rich fibrin (PRF) platelet concentrates forms solid fibrin clots [12]. Injectable PRF (I-PRF), a second-generation platelet concentrate, has attracted attention as a potent and less invasive regenerative agent.

Technically, the non-clotted liquid version of PRF is prepared by low-speed centrifugation [13]. This form of fibrin contains high concentrations of platelets, leukocytes, and growth factors [14]. Since an anticoagulant is not used in the preparation of liquid PRF, it and thrombin are available at the therapeutic injection site, resulting in a slow, gradual release of growth factors. Additionally, I-PRF allows for accurate placement with no morbidity related to the donor site surgery. In a clinical study, Turer et al. observed that gingival recession sites treated with a combination of I-PRF and CTG showed decreased recession depth and increased keratinized tissue height compared with control sites treated with CTG alone [15].

Although numerous clinical studies have highlighted the benefits of I-PRF in combination with regenerative materials for intra-bony defects, limited research has compared the clinical efficiency of the gold-standard CTG placement using the tunnel technique with I-PRF, which is currently considered highly predictable for papilla reconstruction. Hence, the present randomized controlled trial was conducted to compare the effectiveness of I-PRF, delivered by microneedling technique, with that of CTG placement in the management of interdental papilla reconstruction in terms of interdental papilla height (IDPH) and black triangle height (BTH), and also to compare the visual analog scale (VAS) score following the treatment by the two methods.

## Materials and methods

Study design

The present randomized controlled clinical trial was conducted on patients who reported to the outpatient division of a tertiary dental health care center. Initial approval for the study was obtained from the Institutional Ethics Committee of Government Dental College, Kottayam (approval number: IEC/M30/2025/R465/DCK, dated June 17, 2025), and an extension of the approval period for the completion of the project was granted by the Institutional Ethical Committee on June 17, 2025. The study was registered with the Clinical Trials Registry-India (No. CTRI/2024/05/067043). Using G*Power (Heinrich‑Heine‑University Düsseldorf, Düsseldorf, Germany), the sample size was calculated, based on a similar study by Abirami et al. [16], with a power of 80% (α error: 0.05) to detect an effect size of 0.2 (a conservative small-to-medium effect size selection, to ensure adequate power, to detect clinically relevant difference) and the assumption of 20% attrition during the study. A sample size of 30 was obtained, with each group consisting of 15 interventional sites.

The study subjects were aged 18 years and over. They were selected based on the following inclusion criteria: (i) otherwise systemically healthy individuals with at least one Nordland and Tarnow’s class I or class II open gingival or cervical embrasure in the maxillary anterior region, (ii) their selected sites were devoid of any restorations, (iii) the distance from the contact point to the alveolar crest was at least 6 mm by radiographic assessment (i.e., the sites were periodontally healthy), and (iv) their full mouth plaque and bleeding scores were less than 20%. The exclusion criteria were (i) a history of periodontal surgery, (ii) the presence of active orthodontic treatment, (iii) current smokers, (iv) current consumption of any medications that affect periodontal health, and (v) pregnant or lactating women.

**Figure 1:**
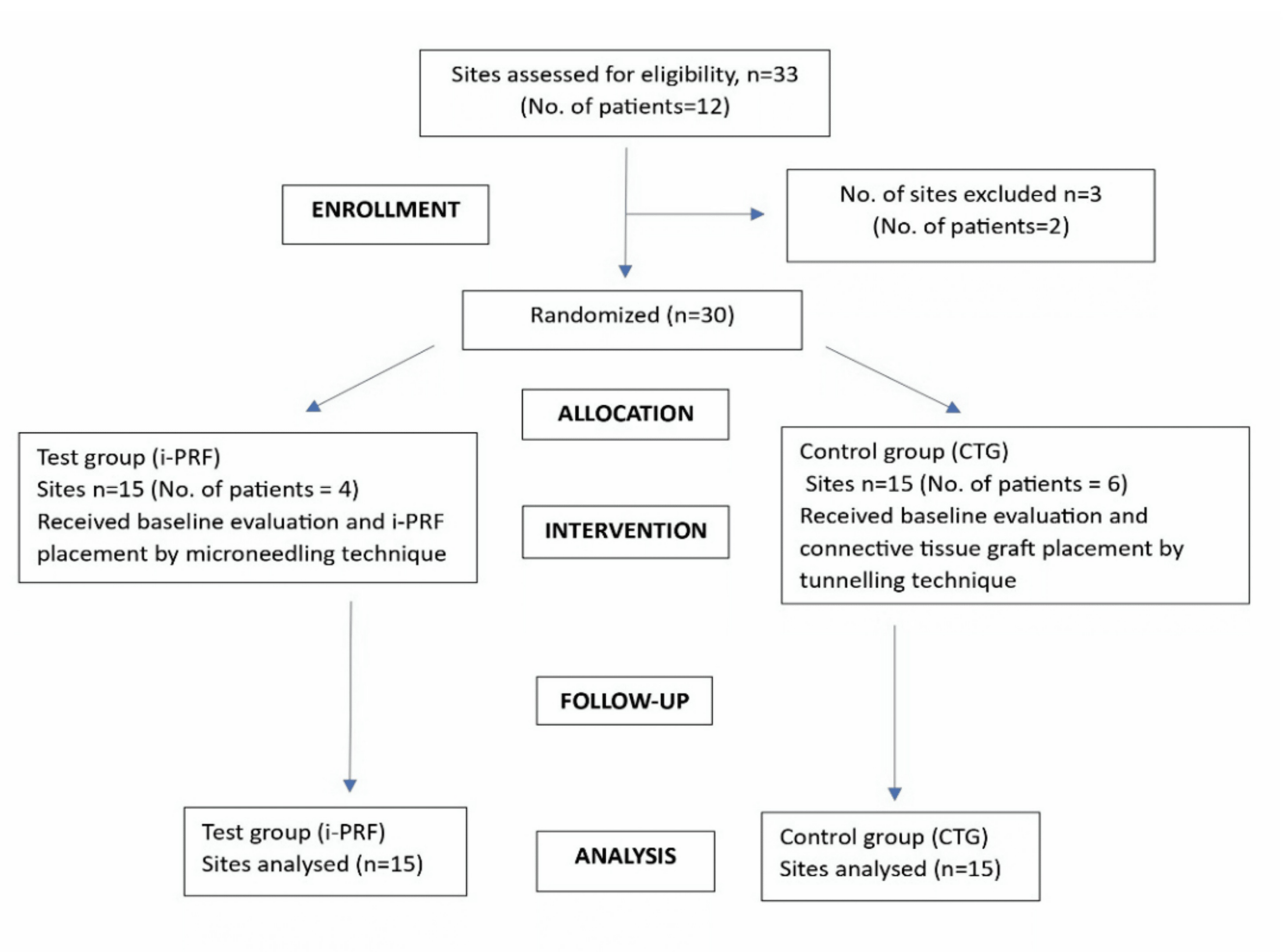
CONSORT flow diagram depicting the methodology, including enrollment, allocation, intervention, follow-up, and analysis of the patients involved in the study No: number, I-PRF: injectable platelet-rich fibrin, CTG: connective tissue graft, CONSORT: Consolidated Standards of Reporting Trials

The participants were randomized to either the test group (the I-PRF group) or the control group (the CTG group) using a blinded computer-generated sequence (Figure 1). Concealed allocation was ensured by using sealed opaque envelopes handled by an individual other than the principal investigator. In accordance with the Declaration of Helsinki (1964; amended 2024), written informed consent was obtained from all of the participants. The subjects’ confidentiality and data protection were strictly maintained throughout the study.

Interventional phase

CTG Group

**Figure 2:**
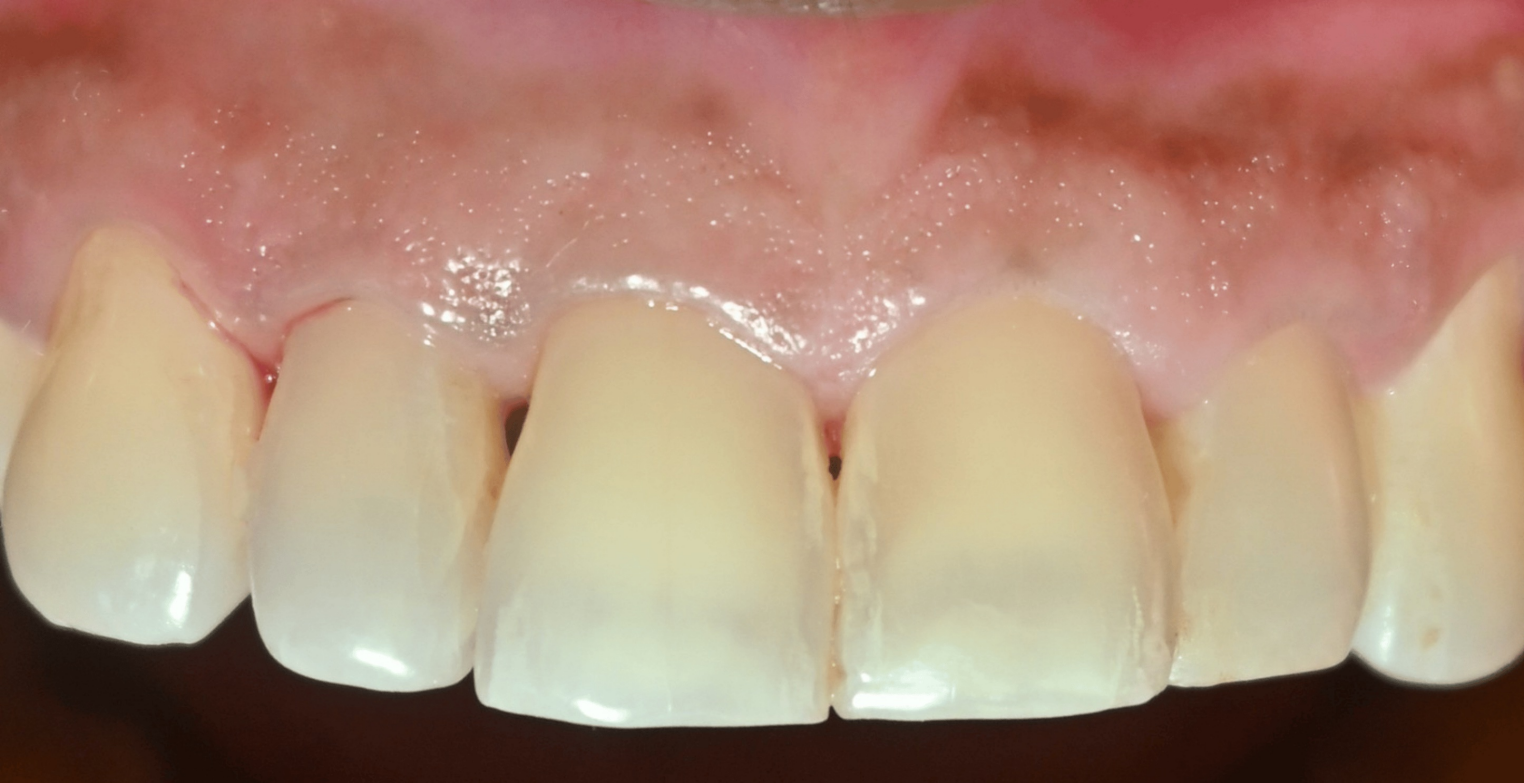
Presence of black triangles between (i) 11 and 21, (ii) 11 and 12, and (iii) 12 and 13

**Figure 3:**
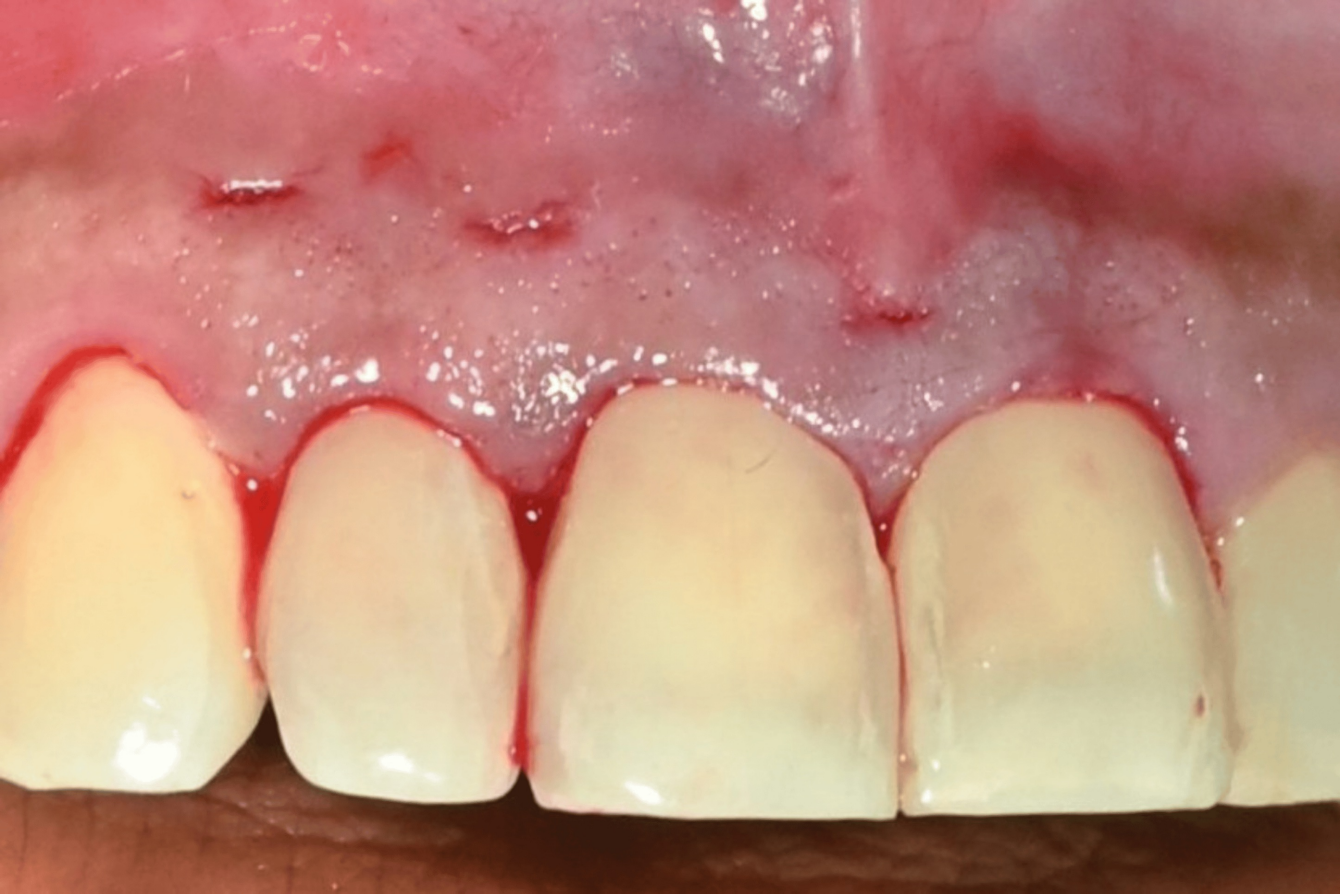
Crevicular and horizontal incisions for tunnel preparation

**Figure 4:**
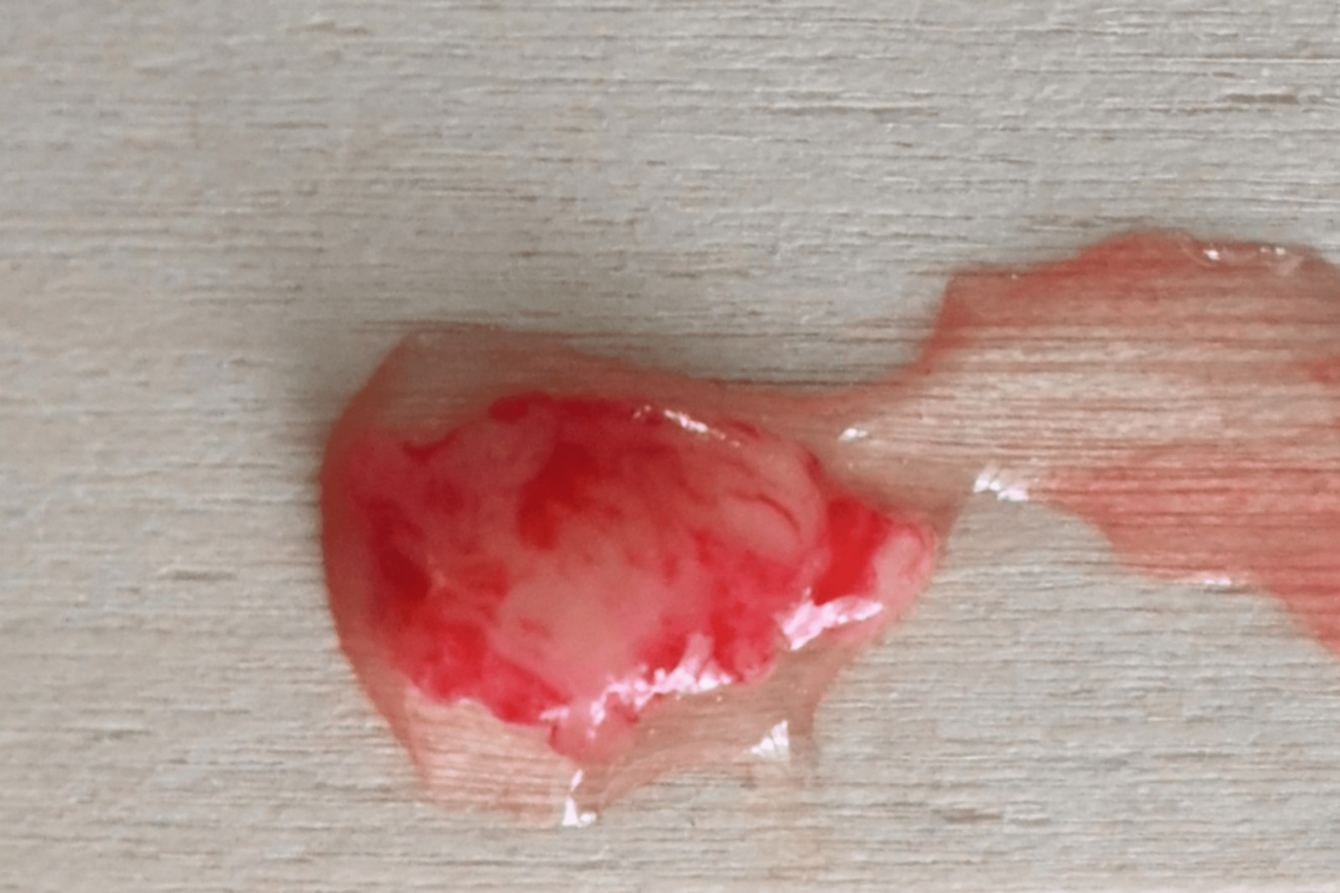
CTG harvested from the palate CTG: connective tissue graft

**Figure 5:**
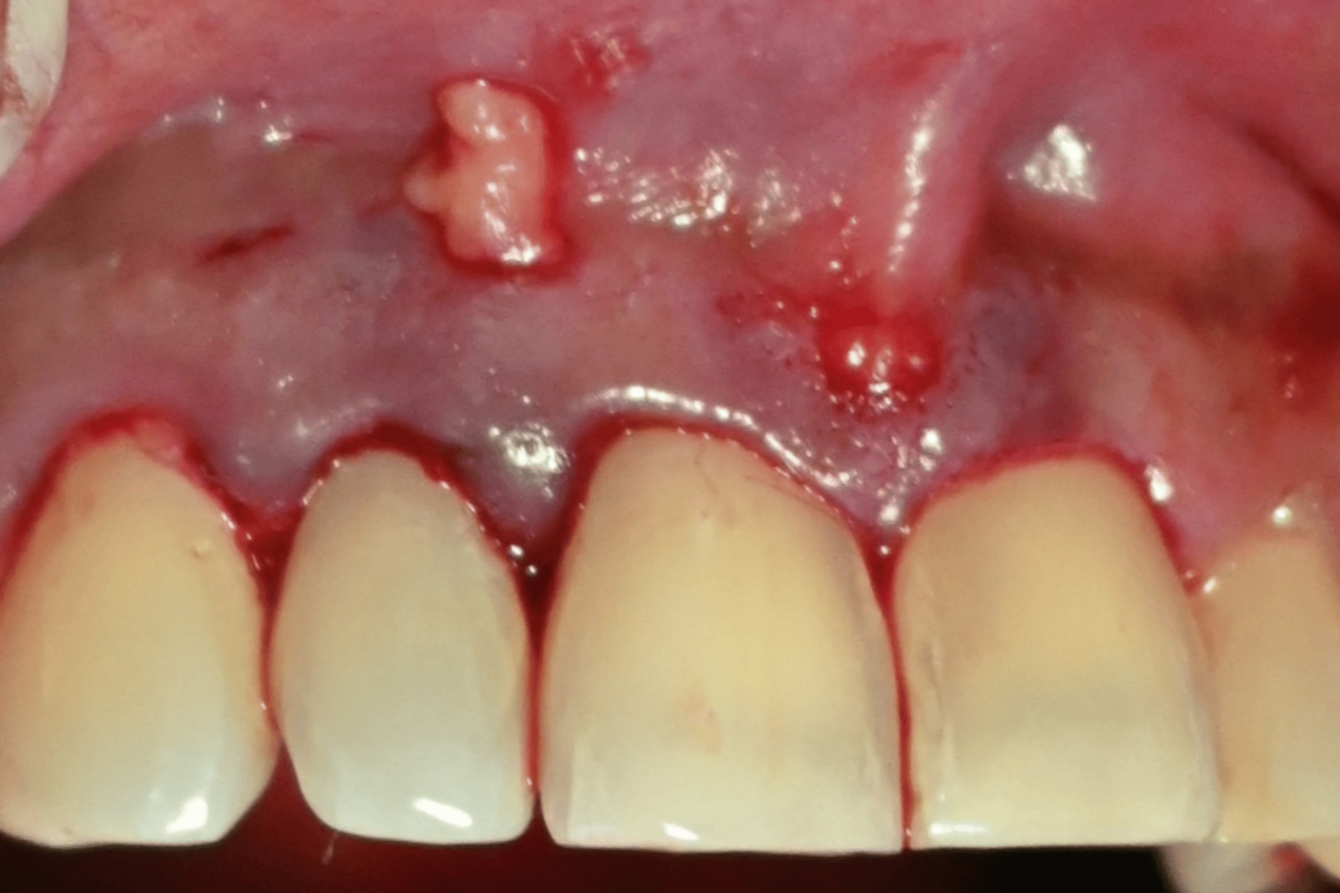
Placement of CTG in the recipient sites CTG: connective tissue graft

**Figure 6:**
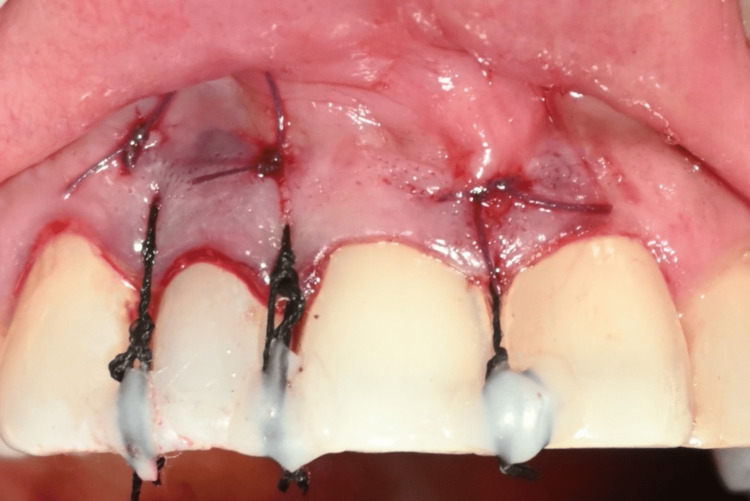
Graft stabilization by suturing with composite stops

CTGs with a uniform thickness of 1.0 mm to 1.5 mm were harvested from the palate using a single-incision technique. At the donor site, the partial-thickness flap was repositioned and secured in place with interrupted sutures (non-resorbable 3-0 Ethilon®, Johnson and Johnson Pvt Ltd). At the recipient site, a full-thickness horizontal incision was made at the level of the mucogingival junction in the buccal aspect and at the level of 2 mm to 3 mm above the papillary base in the palatal aspect, which was no more than 2 mm to 3 mm wide. A crevicular incision was made along the buccal and palatal regions of the adjacent teeth without splitting the interdental gingiva. The tunnel was prepared on the buccal and palatal aspects, extending from the horizontal incision to the interdental area. After placement of the CTG in the tunnel, a horizontal mattress suture (resorbable 5-0 Mendcryl, Sugii Surgical India Pvt Ltd) was placed on the buccal and palatal aspects to stabilize the graft. A vertical mattress suture (non-resorbable 3-0 Ethilon®, Johnson and Johnson Pvt Ltd) was placed on the interdental papilla and suspended on the composite resin placed adjacent to the coronal point of the contact area. The surgical sites were covered with periodontal dressing (CoePakTM GC, IL). After surgery, patients were advised to take analgesia (400 mg ibuprofen, Abbott India Ltd) as needed. All sutures were removed after two weeks (Figures 2-6).

I-PRF Group

**Figure 7:**
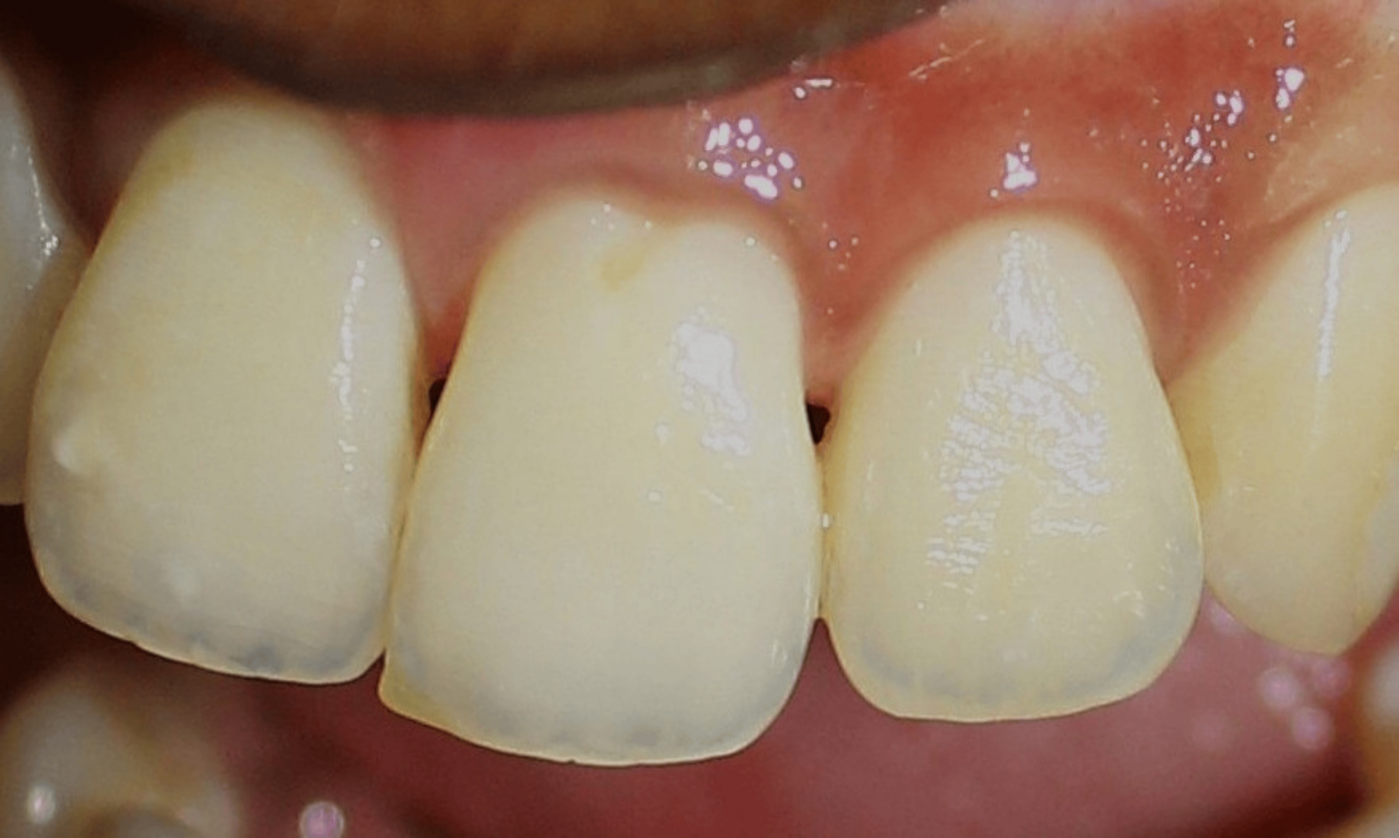
Presence of black triangles between (i) 21 and 22 and (ii) 11 and 21

**Figure 8:**
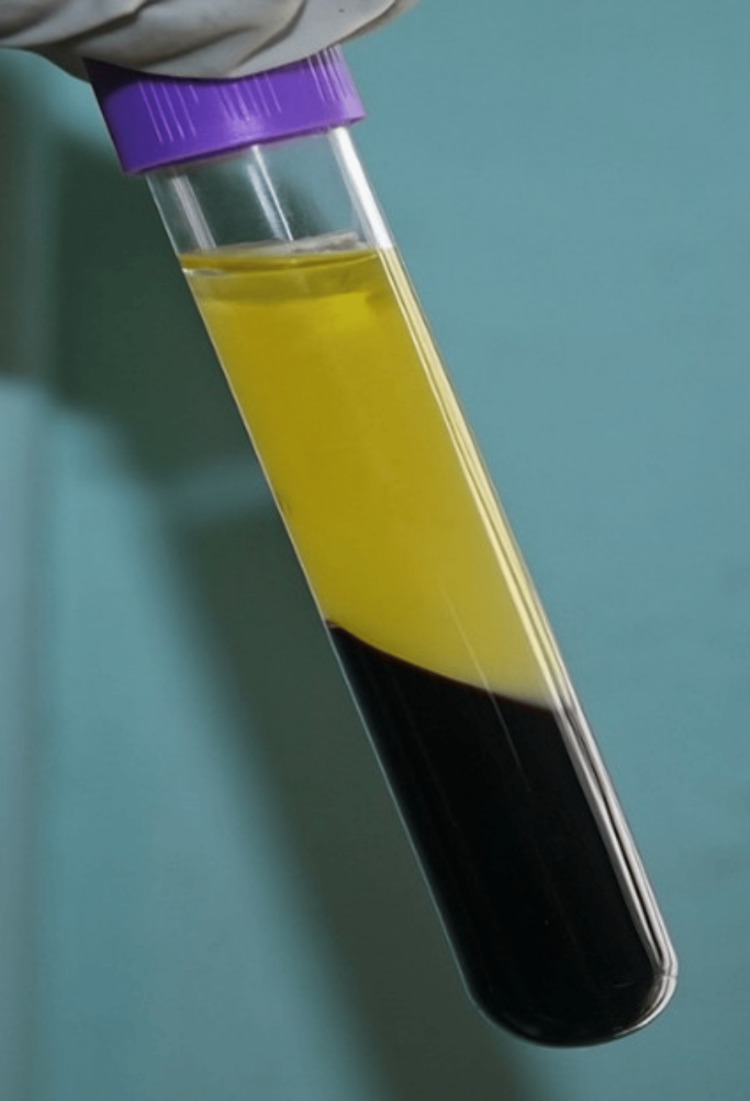
I-PRF on the top layer in the centrifuge tube

**Figure 9:**
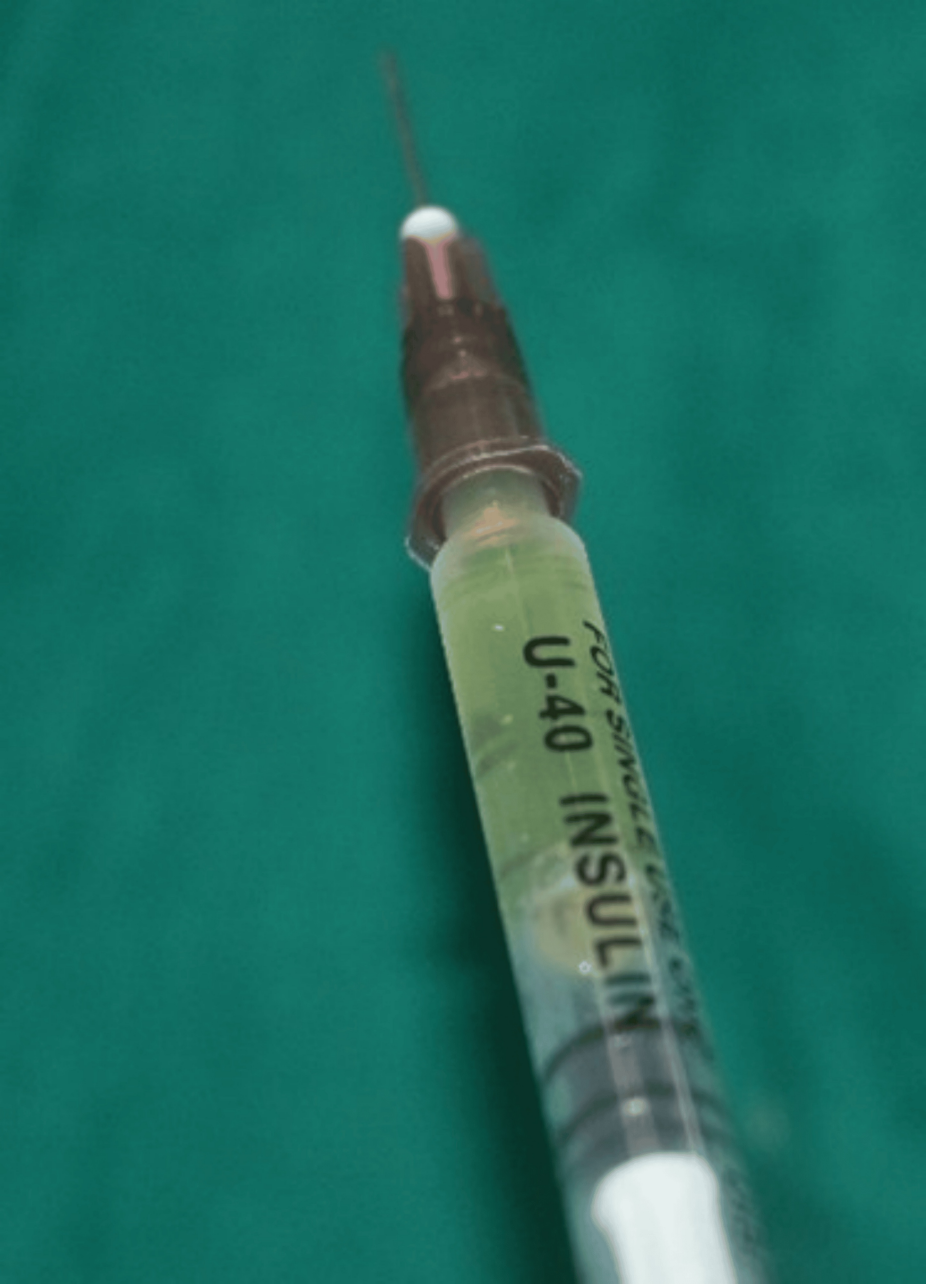
Microsyringe loaded with I-PRF I-PRF: injectable platelet-rich fibrin

To prepare the I-PRF for the test group, 10 ml of each patient’s whole blood without an anticoagulant was centrifuged in a plastic tube at 700 rpm for three minutes in a Choukroun centrifuge (DUO Quattro, LifeCare Devices®, India) with a relative centrifugal force of 55 g and an r-max of 110 mm. About 1 ml of I-PRF from the top layer was loaded into an insulin syringe. The microsyringe needle (needle gauge of 29G) was inserted 2 mm to 3 mm apically to the papilla tip at a 45° angle (with the bevel facing apically). The injections continued until blanching occurred and were followed by a gentle incisal massage with gauze. The patients were advised to take analgesia (400 mg ibuprofen, Abbott India Ltd) as needed during the postoperative period (Figures 7-9).

Clinical assessment

**Figure 10:**
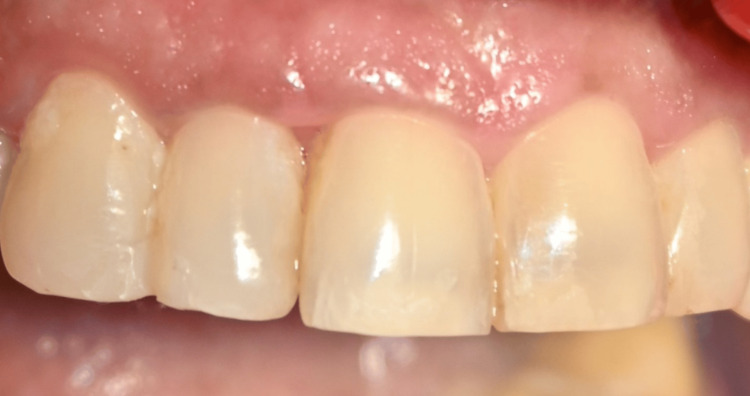
Postoperative picture at one month showing closure of black triangles in the CTG group CTG: connective tissue graft

**Figure 11:**
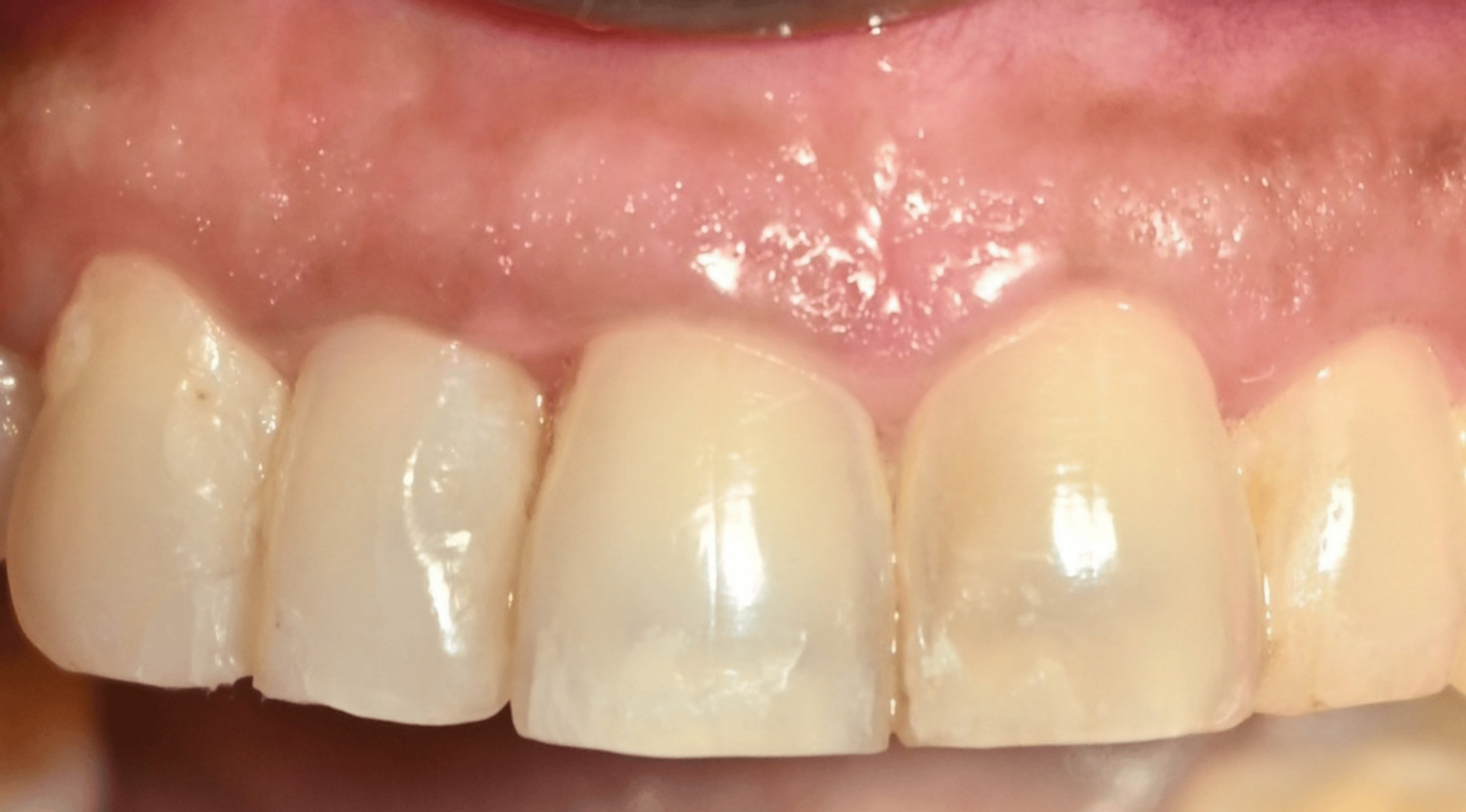
Postoperative picture at three months showing stability of papilla augmentation in the CTG group CTG: connective tissue graft

**Figure 12:**
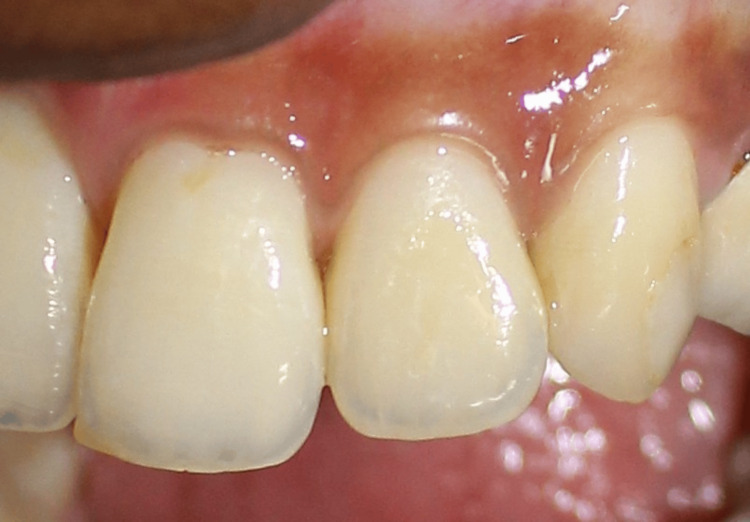
Postoperative picture at one month showing closure of black triangles in the I-PRF group I-PRF: injectable platelet-rich fibrin

**Figure 13:**
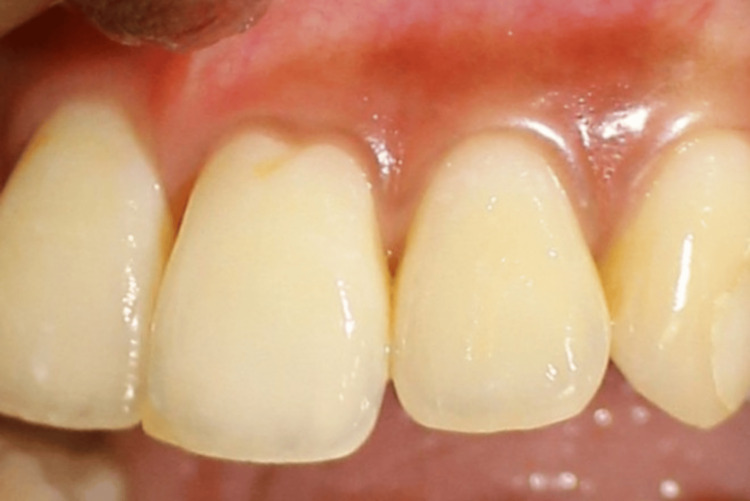
Postoperative picture at three months showing stability of papilla augmentation in the I-PRF group I-PRF: injectable platelet-rich fibrin

The clinical measurements using a UNC-15 probe (Hu-Friedy, Chicago, IL) were recorded by a single masked examiner (BRM). The examiner calibration was performed to record clinical parameters, with an intra-examiner agreement (Kappa 0.86). Customized acrylic stents were used to uniformly reproduce the position of the periodontal probe. The measured parameters consisted of the BTH, which was measured from the tip of the papilla to the apical extent of the interdental contact point; the IDPH, which was measured from a horizontal line connecting the gingival zenith of the two adjacent teeth and the tip of the interdental papilla; the probing pocket depth (PPD); and the clinical attachment level (CAL). Additionally, the full-mouth bleeding score (FMBS) and full-mouth plaque score (FMPS) were recorded. All measurements were performed at baseline (B0), one month (1M), and three months (3M) after treatment (Figures 10-13).

VAS Scoring

The VAS [17], which measures self-reported pain on a 10 cm line from 0 (“no pain”) to 10 (“worst pain”), was used to assess the participants’ pain. The distance from the left end to the patient’s mark indicates the intensity of pain. In this study, the VAS scores were recorded on the third and seventh postoperative days.

Statistical analysis

The data were entered into Excel (Microsoft Corp., Redmond, WA, USA) and analyzed using SPSS Statistics version 30.0 (IBM Corp. Released 2025. IBM SPSS Statistics for Windows, Version 30.0. Armonk, NY: IBM Corp.). The statistical significance was set at p < 0.05 with 95% confidence. Since the data did not follow a normal distribution according to the Shapiro-Wilk test, nonparametric tests were conducted. The Mann-Whitney U test assessed intergroup differences, and the Wilcoxon signed-rank test and Friedman test assessed changes over time within groups.

## Results

In the present study, 30 sites (15 sites per group) in 10 patients were treated. In both groups, all clinical parameters were recorded at B0, 1M, and 3M, while VAS scores were recorded on the third and seventh postoperative days. Nonparametric tests were used for analysis because the normality test indicated nonnormality.

Analysis of primary outcome variables

Table 1 shows the inter- and intra-group comparisons of the mean values of the recorded parameters. At B0, both groups had comparable values for all recorded parameters, as indicated by the Mann-Whitney U test, which yielded a statistically nonsignificant result. All the patients showed minimal and stable FMPS and FMBS values throughout the study period. Correspondingly, the periodontal health parameters, such as PPD and CAL, were also minimal.

**Table 1:** Comparison of the mean values of the clinical parameters over time #: intra-group comparison using the Friedman test, ##: inter-group comparison using the Mann-Whitney U test, *: statistically significant at p < 0.05, SD: standard deviation, mm: millimeters, FMPS: full-mouth plaque score, FMBS: full-mouth bleeding score, BTH: black triangle height, IDPH: interdental papillary height, PPD: probing pocket depth, CAL: clinical attachment level, CTG: connective tissue graft, I-PRF: injectable platelet-rich fibrin

Parameter	Group	Time period			Intra-group analysis^#^ (p-value* and test statistics value)
		Baseline (mean ± SD)	One month (mean ± SD)	Three months (mean ± SD)	
FMPS (%)	CTG	16.73 ± 1.75	14.73 ± 0.88	12.93 ± 1.66	<0.05* (26.528)
	I-PRF	18.07 ± 0.25	15.40 ± 1.05	13.47 ± 0.91	<0.05* (28.182)
	Inter-group analysis^##^ (p-value* and test statistics value)	<0.05* (56.000)	0.073 (73.000)	0.097 (76.500)	
FMBS (%)	CTG	17.50 ± 1.22	15.33 ± 1.03	12.67 ± 1.63	<0.05* (11.565)
	I-PRF	17.00 ± 1.15	15.00 ± 1.15	13.00 ± 2.00	<0.05* (7.000)
	Inter-group analysis^##^ (p-value* and test statistics value)	0.48 (9.000)	0.61 (10.000)	0.43 (8.500)	
BTH (mm)	CTG	2.13 ± 0.64	0.73 ± 0.70	0.80 ± 0.67	<0.001* (29.391)
	I-PRF	2.13 ± 0.83	0.93 ± 0.70	0.87 ± 0.74	<0.001* (29.391)
	Inter-group analysis^##^ (p-value* and test statistics value)	1.00 (112.500)	0.42 (95.000)	0.82 (107.500)	
IDPH (mm)	CTG	3.20 ± 0.77	4.53 ± 0.64	4.53 ± 0.64	<0.001* (30.000)
	I-PRF	3.73 ± 0.79	4.93 ± 0.96	5.00 ± 1.00	<0.001* (29.391)
	Inter-group analysis^##^ (p-value* and test statistics value)	0.08 (73.500)	0.17 (81.500)	0.12 (78.000)	
PPD (mm)	CTG	1.53 ± 0.51	1.13 ± 0.35	1.07 ± 0.25	0.002* (12.286)
	I-PRF	1.47 ± 0.51	1.20 ± 0.41	1.20 ± 0.41	<0.001* (8.000)
	Inter-group analysis^##^ (p-value* and test statistics value)	0.72 (105.000)	0.63 (105.000)	0.29 (97.500)	
CAL (mm)	CTG	2.87 ± 0.35	1.07 ± 0.25	1.07 ± 0.25	<0.001* (30.000)
	I-PRF	2.93 ± 0.25	1.27 ± 0.45	1.27 ± 0.45	<0.001* (30.000)
	Inter-group analysis^##^ (p-value* and test statistics value)	0.55 (105.000)	0.14 (90.000)	0.14 (90.000)	

Comparison of values for each parameter at 1M was performed within each group and between groups. BTH and IDPH were reduced from B0 to 1M in both groups. Considering the individual group, this change was statistically significant. However, intergroup comparison did not reveal a significant difference. In the CTG group, BTH reduced from the mean value of 2.13 ± 0.64 from B0 to 0.73 ± 0.70 at 1M, while in the i-PRF group, the baseline value of 2.13 ± 0.83 reduced to 0.93 ± 0.70. The IDPH value showed a significant increase to 4.53 ± 0.64 at 1M from the B0 value of 3.20 ± 0.77 in the CTG group, with a similar significant improvement in the i-PRF group (3.73 ± 0.79 at B0 and 4.93 ± 0.96 at 1M). On the other hand, no significant difference between the groups was noted at 1M for both parameters.

Statistical analysis of BTH and IDPH values at 3M, compared with B0, showed a significant difference in the intra-group comparison and a nonsignificant difference between groups. In the CTG group, BTH at 1M (0.73 ± 0.70) did not reduce further at 3M (0.80 ± 0.67), while in the i-PRF group, BTH showed further reduction (0.93 ± 0.73 at 1M to 0.87 ± 0.74 at 3M). Interestingly, in the control group, mean IDPH at 1M and 3M were similar (4.53 ± 0.64), whereas IDPH at 3M was higher (5.00 ± 1.00) than at 1M (4.93 ± 0.96) in the test group.

The comparison of changes in clinical parameters over the study period (from B0 to 3M) showed no significant difference between the groups (Table 2). From B0 to 1M, both groups showed similar changes in papillary dimensions, with mean changes of 1.33 ± 0.48 in BTH and 1.20 ± 0.41 in IDPH. However, intergroup comparison was not statistically significant. A similar change was observed by 3M in both groups relative to B0. In comparison, two groups showed a statistically nonsignificant mean change of 1.33 ± 0.48 in BTH and 1.27 ± 0.45 in IDPH from B0 to 3M.

**Table 2:** Inter-group comparison of the changes in the clinical parameters during the study period ##: inter-group comparison using the Mann-Whitney U test, *: statistically significant at p < 0.05, SD: standard deviation, BTH: black triangle height, IDPH: interdental papillary height, PPD: probing pocket depth, CAL: clinical attachment level, CTG: connective tissue graft, I-PRF: injectable platelet-rich fibrin

Parameter	Group	Time period		
		Baseline to one month (mean ± SD)	One month to three months (mean ± SD)	Baseline to three months (mean ± SD)
BTH	CTG	1.33 ± 0.48	0.00 ± 0.00	1.33 ± 0.48
	I-PRF	1.20 ± 0.41	0.17 ± 0.25	1.27 ± 0.45
	Inter-group analysis^##^ (p-value* and test statistics value)	0.011 (97.500)	0.317 (105.000)	0.695 (112.500)
IDPH	CTG	1.53 ± 0.48	0.00 ± 0.00	1.33 ± 0.48
	I-PRF	1.20 ± 0.41	0.17 ± 0.25	1.33 ± 0.48
	Inter-group analysis^##^ (p-value* and test statistics value)	0.010 (97.500)	0.317 (105.000)	0.695 (105.000)
PPD	CTG	0.40 ± 0.50	0.00 ± 0.00	0.47 ± 0.51
	I-PRF	0.27 ± 0.45	0.17 ± 0.25	0.27 ± 0.45
	Inter-group analysis^##^ (p-value* and test statistics value)	0.317 (97.500)	0.317 (112.500)	0.264 (90.000)
CAL	CTG	1.80 ± 0.41	0.00 ± 0.00	1.80 ± 0.41
	I-PRF	1.67 ± 0.48	0.17 ± 0.25	1.67 ± 0.48
	Inter-group analysis^##^ (p-value* and test statistics value)	0.317 (105.000)	0.317 (112.500)	0.417 (97.500)

The comparative analysis of primary outcome variables, i.e., BTH and IDPH, within each study group (intra-group comparison) at both recall examinations (1M and 3M) relative to baseline values showed statistically significant changes. However, these changes were not significant between the test and control groups (inter-group comparison) during the entire evaluation period.

Analysis of secondary outcome variables

Between the study groups, no significant difference was found in the secondary outcome (VAS rates). Nevertheless, VAS scores had reduced in both groups over the study period (Table 3). Even though the comparative analysis of VAS scores between the groups at the third day and seventh day was statistically nonsignificant, the mean change in VAS scores in the CTG group was minimally higher (2.67 ± 1.36) than in the i-PRF group (2.00 ± 0.81). Another secondary outcome assessment was the number of analgesics consumed by the participants. There was a significant difference (p < 0.05) in the mean number of tablets consumed between the test and control groups on both the third and seventh days of the postoperative period. In the CTG group, the mean number of analgesics consumed on the third and seventh days was 4.17 ± 1.72 and 1.83 ± 0.75, respectively, whereas in the i-PRF group, it was 1.75 ± 0.50 on the third day and 0.25 ± 0.50 on the seventh day (not listed in the table).

**Table 3:** Comparison of the mean values of the VAS scores on the third and seventh postoperative days #: intra-group comparison measured using the Wilcoxon signed-rank test, ##: inter-group comparison measured using the Mann-Whitney U test, *: statistically significant at p < 0.05, CTG: connective tissue graft, I-PRF: injectable platelet-rich fibrin, VAS: visual analog scale

Group	Evaluation day		Intra-group analysis^#^ (p-value* and test statistics value)	Mean change (mean ± SD)
	Third day (mean ± SD)	Seventh day (mean ± SD)		
CTG	3.00 ± 1.26	0.33 ± 0.51	0.026 (-2.220)	2.67 ± 1.36
I-PRF	2.00 ± 0.81	0.00 ± 0.00	0.066 (-2.500)	2.00 ± 0.81
Inter-group analysis^##^ (p-value* and test statistics value)	0.187 (6.000)	0.221 (8.000)		

## Discussion

The complex and unique anatomical architecture of the interdental papilla poses challenges for periodontists in achieving predictable results when correcting the unesthetic “black triangles” associated with loss of papillary tissue. A recent systematic review by Patel et al. concluded that sub-epithelial CTGs had the most consistent long-term success in correcting interproximal tissue deficiencies [3]. Additionally, the introduction of the tunneling technique has improved the survival and predictability of grafts [16,18]. Recently, the use of various biomaterials as soft-tissue substitutes has also been shown to be effective [19].

The present study compared the efficiency of CTGs using the tunneling technique with that of the I-PRF microneedling method in papilla reconstruction. Clinically, across all 30 treated sites, both procedures improved papillary dimensions as measured by the IDPH. Similar outcomes of soft tissue graft and platelet concentrates for papilla reconstruction have been previously reported [20,21]. The autogenous nature of biological tissues and the “pedicle graft blood supply” principle of CTGs and I-PRF, which contain an array of bioactive growth factors, make both techniques promising [22].

The superior IDPH values in the CTG group were stable at 1M and 3M, whereas in the I-PRF group, progressive though minimal improvement was observed over the same time period. This improvement was associated with a decreased centrifugation protocol, resulting in an increased number of leukocytes in the top one-third of the platelet concentrates in I-PRF. Thus, there is an increased release of various growth factors within the fibrin matrix, which increases the mRNA expression of transforming growth factor β, platelet-derived growth factor, and COL1a2 [23].

Notable findings were a decrease in PPD and an increase in CAL in both intervention groups. These results are attributable to the significant improvement in gingival inflammatory parameters (i.e., FMPS and FMBS) following the affirmation of oral hygiene care during the review periods, thereby affecting the stability of the regenerated tissues.

The successful regeneration of IDP was achieved in the present study with a single application of I-PRF, a result similar to those reported in other studies [24,25]. However, in the case series by Salkhadi et al., the papillary dimensions did not improve concomitantly after three sessions of I-PRF administration [2]. This result may have been due to the selection of subjects with a higher grade of papillary defects in the latter study than in the other studies.

Regarding BTH, a primary aesthetic concern for patients in the anterior facial zone, both procedures in this study resulted in a significant reduction. Interestingly, the reduction in BTH differed between the CTG group, which showed a minimal increase at 3M compared with 1M, and the I-PRF group, which showed a progressive reduction at the treated sites. I-PRF has several advantages over CTGs, in particular its ability to direct macrophage proliferation toward the M2 phenotype. This pro-resolution marker plays a pivotal role in healing and tissue regeneration [13]. Additionally, it can be hypothesized that, during healing, the nonsurgical mode of delivery reduces the treatment site's dependence on the blood supply relative to CTGs. This aspect is essential considering the unique vascular network in interdental soft tissue. Hence, I-PRF offers better healing dynamics and optimal clinical outcomes than CTGs.

The injection of various regenerative tissue fillers has shown promising results as a nonsurgical method for papillary regeneration [3]. Prajapati et al. compared the BTH reduction achieved with I-PRF and hyaluronic acid delivered via microneedling at various time points. They found that the former was more effective than the latter for reconstructing the interdental papilla [26]. The results of the present study parallel their results. Two other reports have also documented consistent results up to six months using I-PRF for papilla reconstruction [24,25]. These findings demonstrate the efficacy and clinical stability of the I-PRF approach.

The comparison of changes in clinical parameters from B0 to 3M regarding interproximal soft tissue dimensions showed clinically significant improvements in papillary height and a concomitant reduction in interdental black triangles in both study groups. Notably, the I-PRF group showed progressive changes in papillary dimensions from B0 over to the two follow-up examination periods. Improved collagen type II production was observed from 1M through 3M, similar to results from an animal study, and this improvement offers a plausible biologic explanation for the progressive augmentation of IDPH [14]. However, the finding that the intergroup comparison did not reveal significant variation suggests that both groups had comparable outcomes.

The emotional experience of pain produces responses that influence hormone production, stress levels, and compliance. As expected, the short-term VAS scores for pain intensity among patients who received the nonsurgical I-PRF treatment modality decreased from the third to the seventh day, with lower patient morbidity than among patients who received CTGs. Matthews et al. [27] compared patients’ perceptions of surgical and nonsurgical therapies and observed that those treated with soft tissue grafts experienced the most discomfort. Caution is required when assessing these data, however, because the two procedures differ significantly in the clinical sense.

The outcomes of this study have several implications for the management of interdental papilla deficiency. Both CTG and I-PRF effectively reconstructed the interdental papilla and significantly improved key clinical parameters. While CTGs have been considered the gold standard for soft-tissue augmentation, I-PRF offers a less invasive alternative with comparable efficacy and the added patient benefit of reduced postoperative pain. Furthermore, the ease of preparation and application makes I-PRF a preferred chairside option for clinicians.

The limitations of the study include the short follow-up and the limited sample size. Also, assessment of the tissue phenotype would have provided a greater scope to analyze differences in outcomes between the groups. The randomized study design eliminated concern about selection bias and the creation of comparable groups. To the best of our knowledge, this is the first clinical trial to compare the efficacy of the I-PRF microneedling procedure using the tunneling method to augment interdental papillary height with the efficacy of CTGs.

## Conclusions

Both the CTG and I-PRF approaches resulted in significant improvements in BTH and IDPH measurements. The reconstruction of interdental papilla using I-PRF delivered via noninvasive microneedling yielded clinical outcomes comparable to those of CTGs. In addition to improved patient comfort, delivering I-PRF via microneedling helps eliminate donor site morbidity and postoperative pain, as evidenced by decreased analgesic use. Further multicenter studies involving larger numbers of subjects could assess the potential benefit of I-PRF for the management of interdental papillary destruction.
